# Innovation for the Sake of Innovation? How Does Robotic Hepatectomy Compare to Laparoscopic or Open Resection for HCC—A Systematic Review and Meta-Analysis

**DOI:** 10.3390/cancers14143359

**Published:** 2022-07-11

**Authors:** Anastasia Murtha-Lemekhova, Juri Fuchs, Katrin Hoffmann

**Affiliations:** Department of General, Visceral and Transplantation Surgery, Heidelberg University Hospital, 69120 Heidelberg, Germany; anastasia.lemekhova@med.uni-heidelberg.de (A.M.-L.); juri.fuchs@med.uni-heidelberg.de (J.F.)

**Keywords:** robot-assisted hepatectomy, liver surgery, HCC, meta-analysis

## Abstract

**Simple Summary:**

Robot-assisted surgery has gained popularity in urology and colorectal surgery. Some benefits claimed are less complications and faster recovery due to a gentler approach. We aimed to evaluate current evidence on robot-assisted surgery in HCC resection in comparison to standard approaches—laparoscopic and open resections through a systematic review and meta-analysis. Robot-assisted resection was comparable to standardly utilized methods in terms of complication rates. Major complications occurred less but liver-specific complications, such as liver dysfunction or biliary leakage, were similar in frequency. Prospective studies are lacking but are needed to evaluate which patients would really benefit from robot-assisted liver surgery.

**Abstract:**

Robot-assisted hepatectomy is a novel approach to treat liver tumors. HCC is on the rise as the cause of cancer and mortality and is often preceded by cirrhosis. Robot-assisted hepatectomy has been suggested to offer benefits to cirrhotic patients. We aimed to evaluate current evidence for robot-assisted hepatectomy for HCC and compare it to open and laparoscopic approaches. This systematic review and meta-analysis has been conducted in accordance with most recent PRISMA recommendations and the protocol has been registered at PROSPERO (CRD42022328544). There were no randomized controlled trials available and no study focused on cirrhotic patients exclusively. Robot-assisted hepatectomy was associated with less major complications than the laparoscopic approach, but comparable with open hepatectomy. No difference was seen in overall or minor complications, as well as liver specific or infectious complications. Cumulative survivals were similar in robot-assisted hepatectomy and laparoscopic or open approaches. There is a clear lack of evidence to suggest particular benefits for robot-assisted hepatectomy in cirrhotic patients. Otherwise, the robot-assisted approach has similar complication rates as open or laparoscopic methods. Non-industry driven randomized controlled trials are needed to evaluate the efficacy of robot-assisted liver surgery.

## 1. Introduction

Robotic surgery is the paragon of the amalgamation of engineering and surgical skill. Until fairly recently, robot-assisted surgery was a phantom of the mind, but after the first abdominal procedure in 1997 by a Belgian surgeon, Jaques Himpens, the field of robot-assisted abdominal surgery has greatly expanded [1]. Within a decade, robot-assisted surgery has been piloted for procedures spanning from the undemanding to the highly complex [2]. Despite considerable acquisition and maintenance costs, robotic systems are available in tertiary centers worldwide [3].

Robotic surgery offers certain benefits to the surgeon, including protective ergonomics for musculoskeletal health and the decrease of cognitive fatigue, but evidence on the benefits to the patient is less apparent [4]. Due to increased degrees of freedom and tremor elimination, robot-assisted surgery is speculated to lead to lower conversion rates as complex movements can be performed within a confined space. Emphasized advantages of liver robot-assisted surgery include the lower risk of postoperative ascites, infection and pain [3]. Some authors speculate that robot-assisted hepatectomy is particularly beneficial for HCC patients with cirrhosis [5]. 

With exception of rare genetic cases, HCCs develop in the background of liver disease. Whether due to viral hepatitis, metabolic dysfunction-associated, or alcohol-related liver disease, hepatic parenchyma undergoes structural changes that may range from inflammation, steatosis, fibrosis, to cirrhosis. Although a sequential progression to cirrhosis is not a prerequisite, most non-tumor tissue in HCC patients has some degree of damage. A global problem on the rise is a metabolic dysfunction-associated liver disease (MAFLD), which is associated with an increased risk of post-hepatectomy liver failure (PHLF) in patients undergoing liver resection [6]. Cirrhosis is already an established factor for PHLF [7]. With the continued rise of liver disease and the high prevalence of liver cancer, the importance of strategies for curative treatment are rising as well. Surgeons must balance the risk of PHLF and the benefit of hepatectomy while evaluating patients suitable for surgery. Expanding the techniques available and offering the safest treatment options, even at potentially higher material and maintenance costs, is a priority in hepatobiliary surgery. The aim of this systematic review and meta-analysis was to compare the outcomes of robot-assisted hepatectomy (RAH) to open (OH) and laparoscopic (LH) approaches in HCC patients based on current available studies. 

## 2. Materials and Methods

The systematic review and meta-analysis has been conceptualized and is reported in accordance with current PRISMA guidelines [8], as well as in the recommendations of the Cochrane Handbook for Systematic Reviews and Interventions [9]. The protocol has been registered on the PROSPERO international prospective register prior to data extraction (PROSPERO 2022 CRD42022328544). The following PICOS criteria were defined:

Population: patients undergoing hepatectomy for HCC
Intervention: Robot-assisted hepatectomy
Comparison: Laparoscopic or open hepatectomy
Outcomes: complications (overall, major, minor), PHLF, ascites, biliary leak, hemorrhage, infections, conversion rates, recurrence, overall survival, and recurrence free survival.

Recent recommendations were used for the structured literature search [10]. MEDLINE via PubMed, Web of Science and Cochrane Library were searched for publications on HCC resection with robotic approaches without restriction on language or publication date (see Supplementary File S1 for full search strategy). Reference lists of included studies were hand-searched for potentially relevant publications. All publications with comparative study methodology were included, without restriction on prospective or retrospective design, blinding, or randomization. Communications, comments and letters to the editor, editorials, meeting abstracts, and reviews were excluded. Two independent reviewers (AML and JF) performed the title and abstract screening and subsequent full text review. All disagreements were resolved through discussion and consultation with the third reviewer (KH). 

Extraction of data from included studies was performed by two independent reviewers (AML and JF). For data extraction, a standardized form composed prior to the study was utilized and adjusted after first two data extractions. The following data was documented for each publication: title, authors, country, year of publication, journal, funding, study design, interventions, demographics, and clinical outcomes (conversion and complication rates, post-hepatectomy liver failure, ascites, biliary leakage, bleeding, infections, Clavien-Dindo, mortality, recurrence, overall and recurrence-free survivals).

Meta-analyses were performed using publicly available RStudio software, version 4.0.3. The “Metafor”, “meta”, “ggplot2”, and “survival” packages were used. A random-effects model was used for effect estimates for all outcomes due to the anticipated heterogeneity in methodology and clinical framework of relevant studies. Statistical heterogeneity was evaluated using the I^2^ statistics. An I^2^ value below 25% indicated low, and over 75% indicated high heterogeneity. The Mantel-Haenszel method was used for pooling odds ratios and 95% confidence intervals in dichotomous endpoints. Aggregated data was compared between groups. Categorical values were compared with an χ^2^ test, while an independent sample t-test was used for continuous variables. Survival was assessed using the Kaplan-Meier method.

The methodological quality of included studies was evaluated with ROBINS-I [11] and the certainty of evidence was assessed using GRADE [12] for significant outcomes and outcomes reported by three or more studies.

## 3. Results

**Figure 1:**
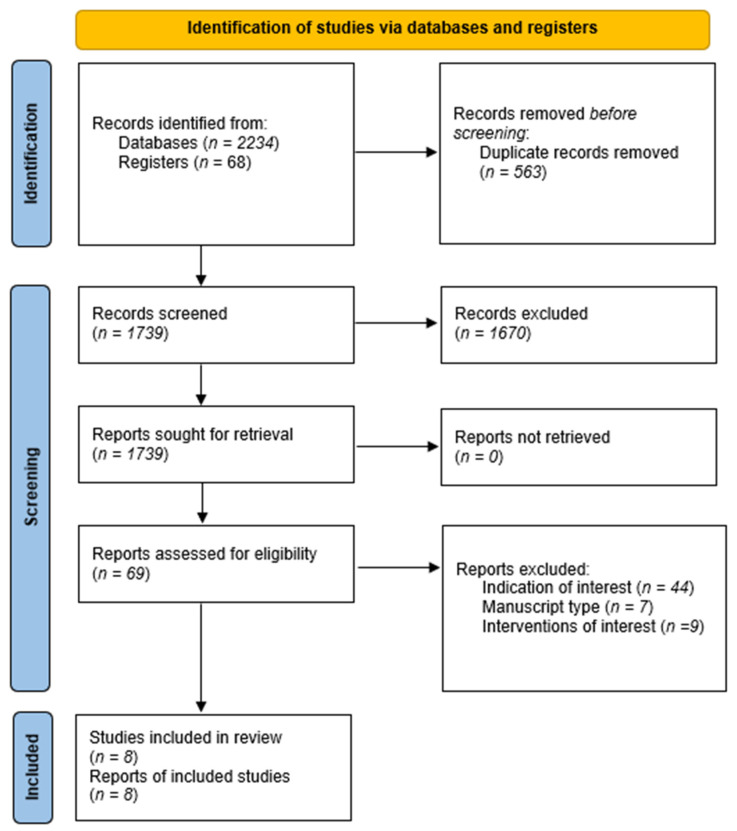
The study selection flow.

After exclusion of duplicates, 1739 records were screened, from which 69 were assessed for eligibility. After exclusion of articles that did not study the indication or intervention of interest, as well as manuscript types, eight studies were included in the analysis. The overview of the study selection process is depicted in Figure 1 and a description of included studies is provided in Table 1.

All studies were of retrospective design; five studies compared RAH to LH [13,15,16,17,18]. A comparison between OH and RAH was done by three studies [14,19,20]. There were no randomized controlled trials available and no study focused on cirrhotic patients exclusively.

### 3.1. Critical Appraisal of Included Studies

The risk of bias was assessed using ROBINS-I and included the assessment of seven domains: bias due to confounding, selection, classification of interventions, missing data, measurement of outcomes, and reported results. Overall, the risk of bias in studies was low (Table 2).

### 3.2. Characteristics of Included Studies and Patients in RAH and LH Comparisons

The five studies comparing RAH and LH had retrospective designs, and only one performed a propensity score matching for the two groups. Overall, 529 patients after LH were compared to 324 patients after RAH (Table 3). The primary identified liver disease in both studies was viral, and the distribution of background liver disease was similar between groups.

Concerning tumor characteristics, only differentiation could be assessed between groups, as no other characteristic was provided by multiple studies. The differentiation of HCC was similar between groups, with G1 in 85 vs. 36, G2 155 vs. 68, and G3/G4 36 vs. 23 cases, in LH and RAH, respectively.

**Figure 2:**
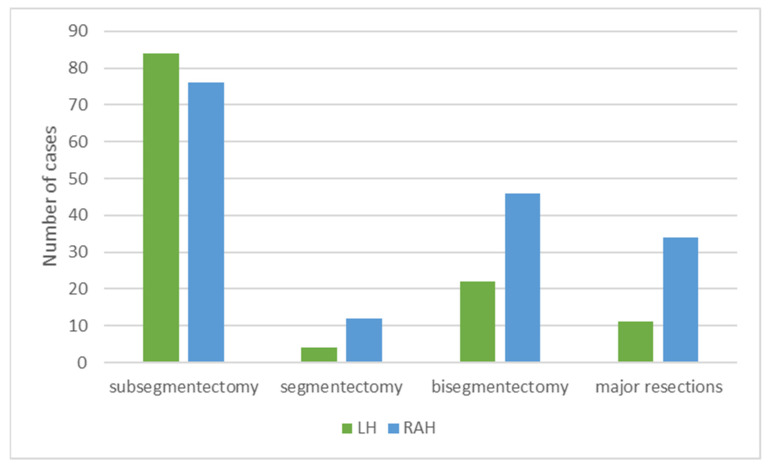
Distribution of surgeries performed.

The distribution of surgeries performed was significantly different [χ^2^ = (1, 368) = 7.5583, *p* = 0.006], as more major resections were performed in the RAH than LH. However, the pooled comparison was based on four studies, while the largest study omitted the data (Figure 2).

### 3.3. Comparison between RAH and LH

**Figure 3:**
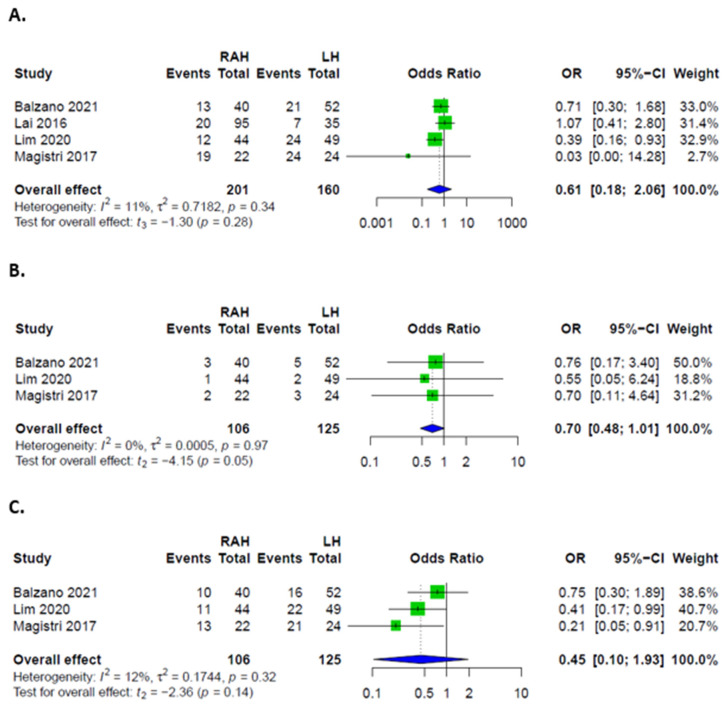
Forest plots for complications; (**A**) Overall complications in patients after RAH and LH; (**B**) Major complications in patients after RAH and LH; (**C**) Minor complications in patients after RAH and LH.

**Figure 4:**
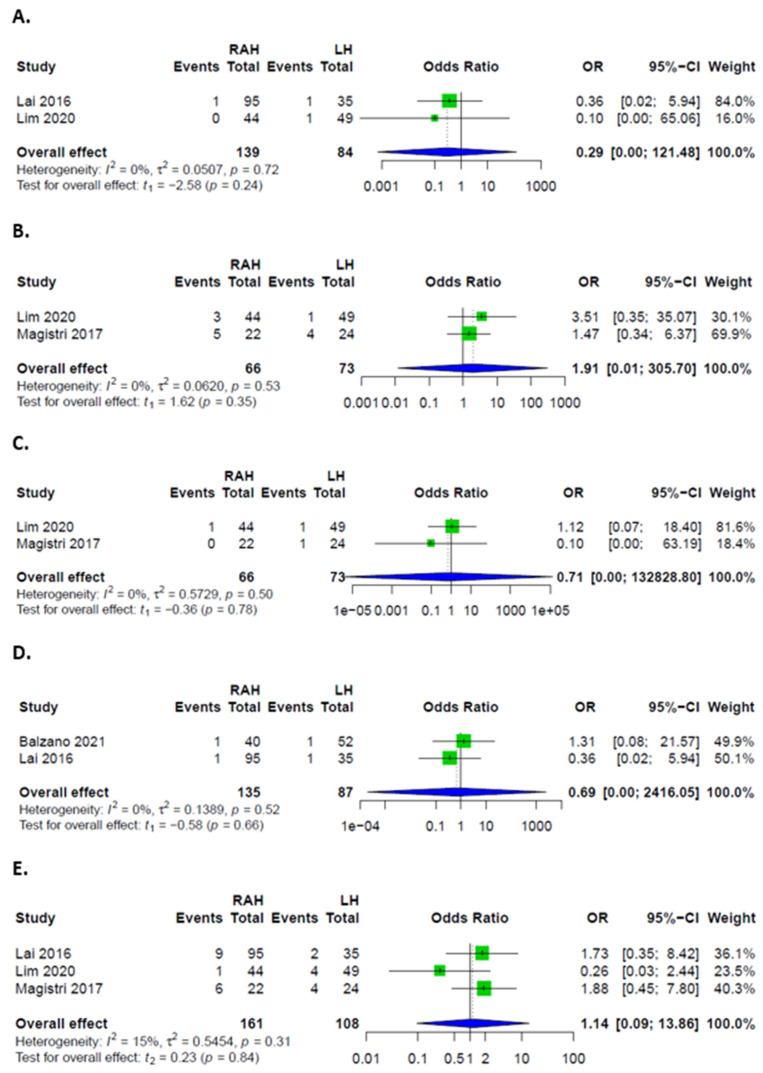
Forest plots for specific complications. (**A**) PHLF in patients after RAH and LH. (**B**) Postoperative ascites in patients after RAH and LH. (**C**) Postoperative biliary leakages in patients after RAH and LH. (**D**) Postoperative hemorrhages in patients after RAH and LH. (**E**) Postoperative infections in patients after RAH and LH.

**Figure 5:**
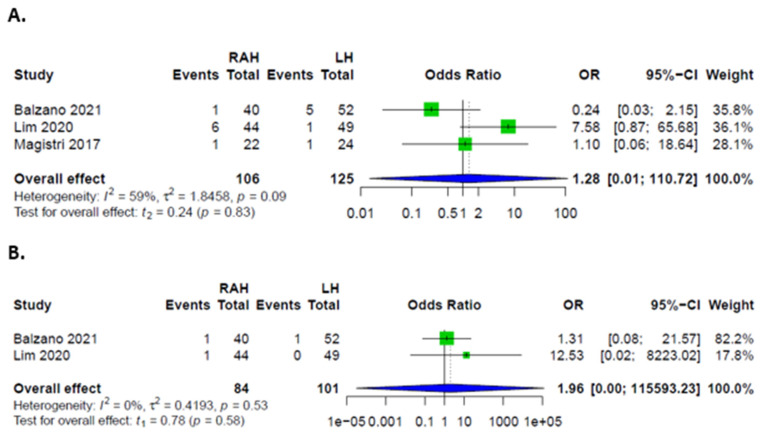
Forest plots for additional interventions; (**A**) Intraoperative transfusions in patients undergoing RAH and LH; (**B**) Repeat surgeries in patients after RAH and LH.

Overall, there were no significant differences in complication rates between RAH and LH. Rates of major complications, defined as Clavien-Dindo grades III and IV, were higher in LH. Patients had similar odds of developing minor complications, as well as PHLF, ascites, biliary leakages, hemorrhages, and infections in both groups (Figure 3 and Figure 4). Rates of intraoperative transfusions and reoperations were similar as well (Figure 5). No mortality was described for either group in the included studies.

**Figure 6:**
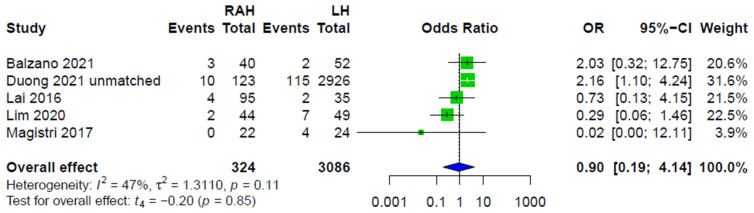
Forest plot for conversion rates during LH and RAH.

All five studies comparing LH to RAH provided data on conversion, and summary effect did not show significant differences between the LH and RAH approaches (Figure 6).

No data was provided to evaluate functional recovery, e.g., mobilization or physical activity levels. Studies did not provide data to evaluate ICU/IMC length of stay.

### 3.4. Oncological Outcome in Patients after RAH and LH Approaches

**Figure 7:**
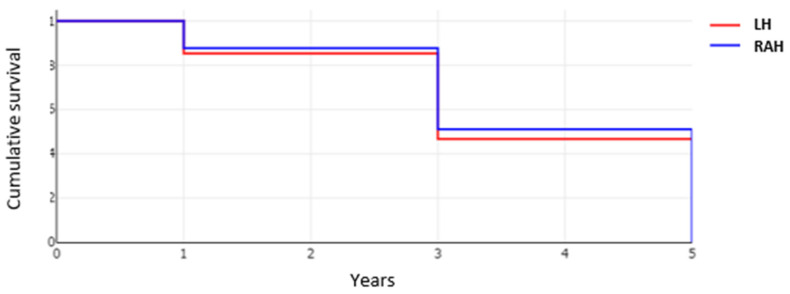
Cumulative survival for LH and RAH approaches.

Clear resection margin (R0) was significantly more often achieved in the RAH group with 96.8% (152 of 157 cases), than in the LH group, with 91.0% (101 of 111 cases) (*p* = 0.041). Recurrence rates were similar in both groups with 35.6% of patients (48 of 135) in the RAH and 46.0% cases (40 of 87) developing a recurrence within up to five years of follow-up (*p* = 0.12). The overall survival after surgery was comparable between the two groups (*p* = 0.769) (Figure 7).

### 3.5. Characteristics of Included Studies and Patients in RAH and OH Comparisons

**Figure 8:**
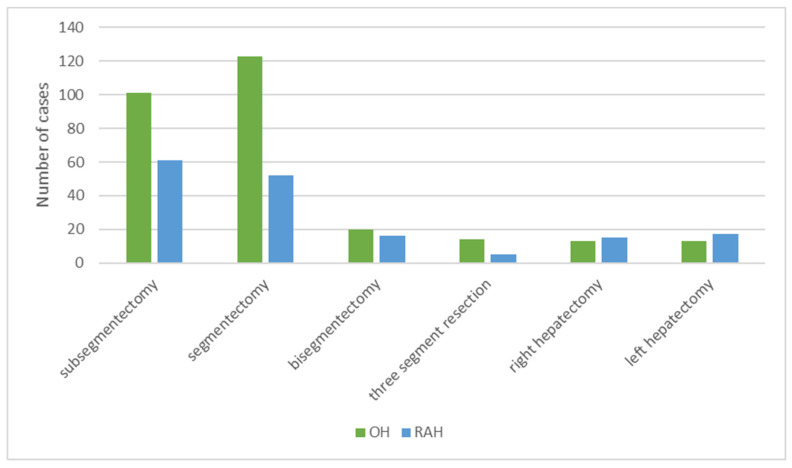
Distribution of surgeries in OH and RAH.

Three retrospective studies, one of them PSM, compared the OH and RAH approaches. An RAH group of 167 was compared against 289 patients in the OH group. Pooled data on patient demographics is provided in Table 4. In total, 244 minor and 45 major surgeries were performed openly and 129 minor and 38 major hepatectomies were robot-assisted (*p* = 0.06) (Figure 8).

### 3.6. Comparison between RAH and OH

**Figure 9:**
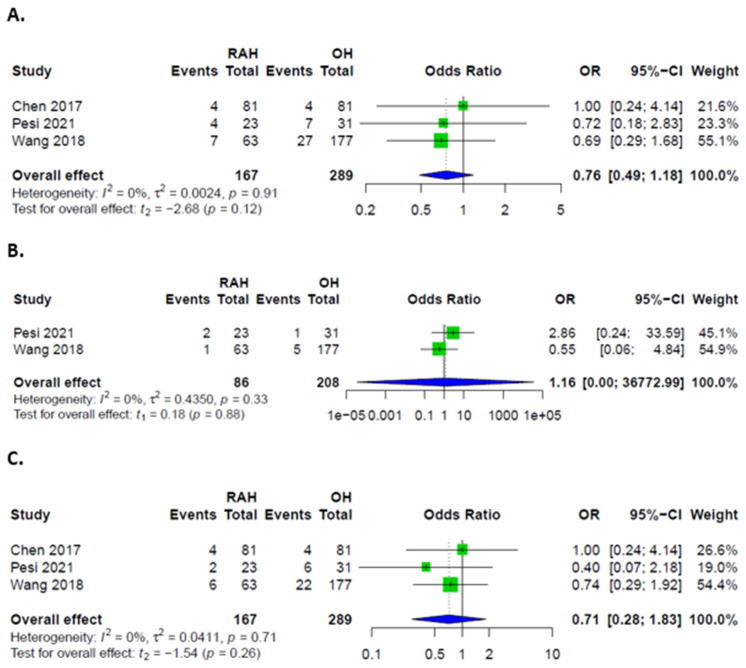
Forest plots for complications; (**A**) Overall complications in patients after RAH and OH; (**B**) Major complications in patients after RAH and OH; (**C**) Minor complications in patients after RAH and OH.

**Figure 10:**
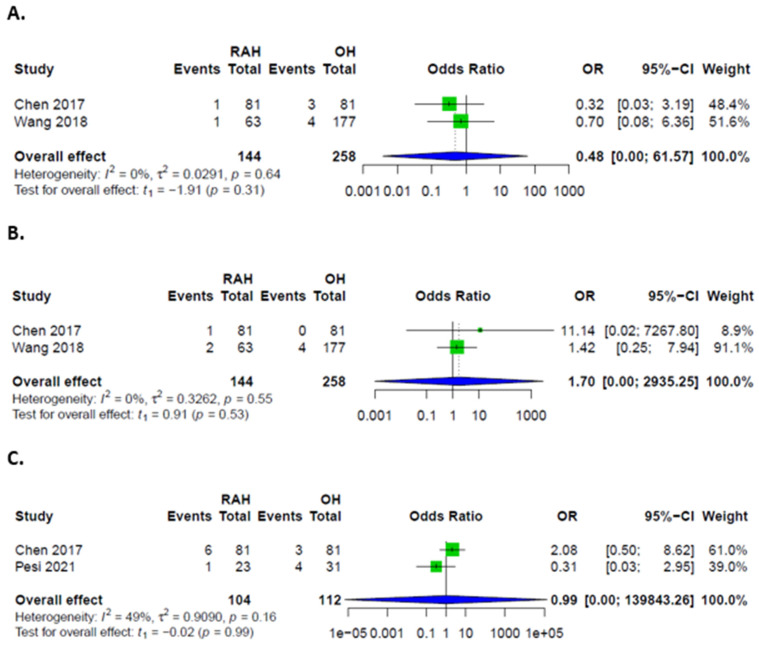
Forest plots for specific complications and interventions; (**A**) Biliary leakages in patients after RAH and OH; (**B**) Postoperative hemorrhages in patients after RAH and OH; (**C**) Intraoperative transfusions in patients after RAH and OH.

Overall, major and minor complications were comparable between the two groups (Figure 9). Specific complications could only be assessed for biliary leakages and hemorrhages, which were also comparable (Figure 10). PHLF was only described in one case, which was resected via the open approach. Ascites was additionally described by one study in 10 cases in the OH and one case in the RAH group. Concerning additional interventions, only sufficient data on transfusions was provided, without significant difference between groups (Figure 10). No data was provided to evaluate functional recovery, e.g., mobilization or physical activity levels. Total hospital stay, as well as ICU/IMC length of stay, could not be pooled for analysis.

### 3.7. Oncological Outcome in Patients after RAH and OH Approaches

**Figure 11:**
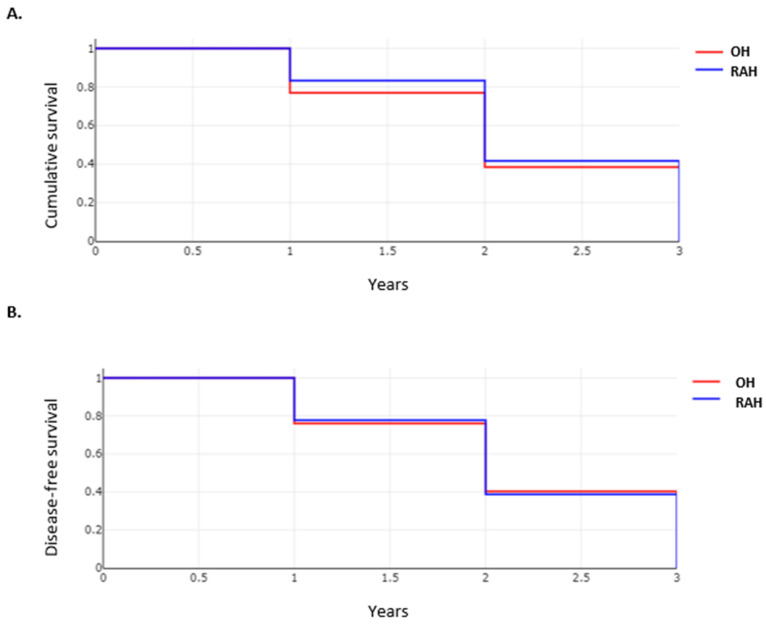
Survivals of patients in OH and RAH groups; (**A**) Overall-survivals of OH and RAH groups; (**B**) Disease-free survival of OH and RAH groups.

Resection margins were comparable between the two groups, with R0 achieved in 97.2% of cases (281 of 289) in the OH group vs. 96.4% cases (161 of 167) in the RAH group (*p* = 0.6). Recurrence rates were only reported in one study, but overall and disease-free survivals were aggregated from two studies and are depicted in Figure 11. No difference was observed in overall and disease-free survivals between the two groups. 

### 3.8. Certainty of Evidence

A Grading of Recommendations Assessment, Development and Evaluation (GRADE) approach was utilized to rate the certainty of evidence. The main outcomes are listed in Table 5. Overall, due to the exclusively retrospective study design and lack of matching in most included studies, the certainty of evidence ranged from low to very low.

## 4. Discussion

Robot-assisted surgery has gained popularity among surgeons and developers, and it is an approach that will change the future of abdominal surgery [3]. The allure is not merely that of novelty but of possibilities. It has the potential to provide access to minimally invasive surgery specialists where the distance would otherwise preclude it [21]. Additionally, due to elegant engineering, robot-assisted surgery is able to offer precision surgery while eliminating imprecision due to hand tremors [22]. Despite potentialities that the surgeons expect, robot-assisted surgery needs meticulous elucidation. Although benefits are suggested in certain patient groups, like cirrhotic patients undergoing liver resection, the evidence to support this claim is clearly lacking [5]. Thus far, studies have not been focused on patients with cirrhosis. Not only did the studies not differ statistically in number of cirrhotic patients undergoing RAH or another approach, it was mostly unclear how cirrhotic patients were identified and defined. Furthermore, there are no randomized controlled trials comparing RAH, LH and open surgery. Additionally, no studies compared material costs in addition to cost and revenue within the reimbursement system. 

With MAFLD rising as the cause of HCC and NAFLD being associated with higher PHLF rates, an interesting question to examine is the influence of steatosis, fibrosis and NASH on outcomes in RAH versus LH or OH. This is particularly relevant, as obesity, frequently concurring in MAFLD patients, may pose an additional limitation for minimally invasive surgery, and the benefits of the RAH approach would be interesting to consider [6].

Although RAH offers some benefits that may potentially lead to lower complication rates, such as enabling elaborate motions in limited space, the current evidence suggests that, so far, RAH is only comparable to LH or OH. In particular, few liver specific complications are reported for either approach, which may indicate that on one hand, hepatectomy has become a fairly safe procedure at hospitals offering state-of-the-art techniques, but also that a wider patient selection is needed to evaluate the approaches. Although functional recovery is often the primary reason for minimally invasive surgery and, more recently, robot-assisted surgery, mobilization, return to physical activity, pain levels, and quality of life has not been reported by studies thus far. Total length of hospital stay is similar for RAH and LH, but whether ICU/IMC observation is comparable as well remains unclear. The lengths of stay are underreported in comparisons between RAH and OH. Our study is the first systematic review to specifically investigate the impact of RAH versus LH or OH in patients with HCC. According to our results, RAH achieves comparable outcomes as LH and OH. Suggested significant benefits for cirrhotic patients were not confirmed by the present meta-analysis due to lack of well-designed clinical trials and real evidence. 

A limitation for the certainty of evidence is the designs of the included studies, as they were all retrospective in nature, with only two studies attempting to match patient groups through propensity-scores [14,18]. Although retrospective evidence suggests that RAH is a robust approach in patients with HCC, prospective studies, in particular RCTs, are needed to truly evaluate whether RAH offers benefits. The largest included retrospective study has omitted the data on procedure distribution, which in turn poses a bias in the comparison of complications [15]. As one of the arguments for RAH is the gentler approach that may reduce liver-specific complications, RCTs will need to be sufficiently powered to evaluate the occurrence of PHLF in patients treated with various approaches. As studies reported so far only included one case of PHLF in patients after RAH versus two in patients after LH, clinicians contemplating an RCTs will need to consider the numbers to treat accordingly. Homogeneity also needs to be considered, and the pooling of major and minor hepatectomies into one RCT should be avoided. Similar to RCTs for colorectal tumors, heterogeneity in tumor stages should also be avoided [23].

## 5. Conclusions

Robot-assisted hepatectomy has similar complication rates as the laparoscopic or open approaches. Major complications may be lower in RAH compared to LH, but more studies are needed to evaluate those in depth. Liver-specific complications have not shown to be reduced in RAH, and there is no evidence to support that patients with cirrhosis in particular should be favored for the robot-assisted approach.

## Figures and Tables

**Table 1:** Overview of included studies.

Publication	Population	Design	*n* (RAH)	*n* (LH)	*n* (OH)
Balzano 2021 [13]	Italy	Retrospective	40	52	
Chen 2017 [14]	Taiwan	Retrospective, PSM	81		81
Duong 2021 [15]	USA	Retrospective, PSM	123	369	
Lai 2016 [16]	China	Retrospective	95	35	
Lim 2020 [17]	France	Retrospective	44	49 (3D)	
Magistri 2017 [18]	Italy	Retrospective	22	24	
Pesi 2021 [19]	Italy	Retrospective	23		31
Wang 2018 [20]	Taiwan	Retrospective	63		177

**Table 2:** ROBINS-I risk of bias assessment for included studies.

	Bias Due to Confounding	Bias in Selection of Participants into the Study	Bias in Classification of Interventions	Bias Due to Deviations from Intended Interventions	Bias Due to Missing Data	Bias in Measurement of Outcomes	Bias in Selection of the Reported Results	Overall
Balzano 2022 [13]	H	L	L	L	L	L	L	L
Chen 2017 [14]	L	L	L	L	L	L	L	L
Duong 2021 [15]	L	L	L	L	M	L	M	L
Lai 2016 [16]	L	L	L	L	L	L	L	L
Lim 2020 [17]	L	M	L	L	M	L	L	L
Magistri 2017 [18]	L	L	L	L	L	L	M	L
Pesi 2021 [19]	L	L	L	L	L	L	M	L
Wang 2018 [20]	L	M	L	L	L	L	L	L

L = Low, M = Moderate.

**Table 3:** Aggregated demographics.

Characteristics	RAH	LH	*p*
	*n =* 324	*n =* 613	
Sex			
Male Female	233 (71.9%)	380 (62.0%)	0.98
	91 (28.1%)	233 (38.0%)	
Age			
Mean ± SD Range	62.5 ± 0.21	63.05 ± 11.44	0.54
	46–83	34–86	
Liver disease			
Alcohol-related Viral MAFLD None Unidentified/other	13 (4.0%)	13 (2.1%)	0.16
	126 (38.9%)	75 (12.2%)	
	9 (2.8%)	12 (2.0%)	
	5 (1.5%)	6 (1.0%)	
	171 (52.8%)	507 (82.7)	
Cirrhosis			
Yes No Unknown	161 (49.7%)	137 (22.3%)	0.27
	40 (12.3%)	107 (17.5%)	
	123 (38.0%)	369 (60.2%)	
Primary surgery			
Yes No Unknown	130 (40.1%)	79 (12.9%)	0.96
	9 (2.8%)	89 (14.5%)	
	185 (57.1%)	445 (72.6%)	
Tumor size			
Mean ± SD Range	35.6 ± 21.3	31.9 ± 18.5	0.14
	12–65	Nov-58	
Surgery time			
Mean ± SD Range	204.4 ± 101.8	212.4 ± 80.4	0.49
	95–390	80–256	
Pringle time			
Mean ± SD	34 ± 20.6	38.8 ± 23.6	0.11
Hospital stay			
Mean ± SD	6.9 ± 4.9	6.7 ± 2.6	0.77

SD: standard deviation.

**Table 4:** Aggregated demographics for RAH versus OH comparison.

Characteristics	RAH	OH	*p*
	*n* = 167	*n* = 289	
Sex			
Male Female Unidentified/other	61 (36.5%)	146 (50.5%)	0.9
	25 (15.0%)	62 (21.5%)	
	81 (48.5%)	81 (28.0%)	
Liver disease			
Alcohol-related Viral MAFLD None Unidentified/Other	2 (1.2%)	3 (1.0%)	0.7
	68 (40.7%)	155 (53.6%)	
	3 (1.8%)	0	
	0	0	
	94 (56.3%)	131 (45.3%)	
Cirrhosis			
Yes No Unknown	54 (32.3%)	64 (22.1%)	0.4
	50 (29.9%)	48 (16.6%)	
	63 (37.7%)	177 (61.2%)	
Child-Pugh Grade			
A B C Unknown	81 (48.5%)	199 (68.8%)	0.8
	2 (1.2%)	4 (1.4%)	
	0	0	
	84 (50.3%)	86 (29.8%)	
Primary surgery			
Yes No Unknown	63 (37.7%)	177 (61.2%)	NA
	0	0	
	104 (62.3%)	112 (38.8%)	
TNM			
I II III Unknown	98 (58.7%)	162 (56.1%)	**0.03**
	42 (25.1%)	70 (24.2%)	
	4 (2.4%)	26 (9.0%)	
	23 (13.8%)	31 (10.7%)	
Vascular invasion			
V0 V1 V2 Unknown	123 (73.7%)	221 (76.5%)	0.9
	39 (23.3%)	68 (23.5%)	
	0	0	
	0	0	

**Table 5:** GRADE.

Outcome	№ of Included Studies	Certainty of the Evidence (GRADE)	Relative Effect (95% CI)
RAH versus LH Overall complications	4 retrospective	Low	OR 0.61 [0.18; 2.06]
RAH versus OH Overall complications	3 retrospective	Very Low	OR 0.76 [0.49; 1.18]
RAH versus LH Major complications	3 retrospective	Very Low	OR 0.70 [0.48; 1.01]
RAH versus LH Minor complications	3 retrospective	Very Low	OR 0.45 [0.10; 1.93]
RAH versus OH Minor complications	3 retrospective	Very Low	OR 0.71 [0.28; 1.83]
RAH versus LH Infections	3 retrospective	Very Low	OR 1.14 [0.09; 13.86]
RAH versus LH Transfusions	3 retrospective	Very Low	OR 1.28 [0.01; 110.72]
RAH versus LH Conversions to OH	5 retrospective	Low	OR 0.90 [0.19; 4.14]

## Data Availability

All data contributing to the analysis of this manuscript is available from the corresponding author upon reasonable request.

## Supplementary Materials

The following supporting information can be downloaded at: https://www.mdpi.com/article/10.3390/cancers14143359/s1, Supplementary File S1: search strategy.
