# Supplementary material for: Innovation for the Sake of Innovation? How Does Robotic Hepatectomy Compare to Laparoscopic or Open Resection for HCC—A Systematic Review and Meta-Analysis
Source: Cancers (Basel). 2022 Jul 11;14(14):3359. doi: 10.3390/cancers14143359 (PMC9318519; doi:10.3390/cancers14143359)
Supplement: Supplementary file 1 [file cancers-14-03359-s001.zip › cancers-1812733-supplementary.pdf]

Medline Search strategy:

"robot*"[All Fields] AND ("liver"[MeSH Terms] OR "liver"[All Fields] OR "livers"[All Fields] OR "liver s"[All Fields] OR ("hepatectomy"[MeSH Terms] OR "hepatectomy"[All Fields] OR "hepatectomies"[All Fields]))
